# The Effect of Osteopathic Visceral Manipulation on Quality of Life and Postural Stability in Women with Endometriosis and Women with Pelvic Organ Prolapse: A Non-Controlled Before–After Clinical Study

**DOI:** 10.3390/jcm14030767

**Published:** 2025-01-24

**Authors:** Małgorzata Wójcik, Małgorzata Kampioni, Zuzana Hudáková, Idzi Siatkowski, Witold Kędzia, Grażyna Jarząbek-Bielecka

**Affiliations:** 1 Department of Physiotherapy, Faculty of Physical Culture in Gorzow Wielkopolski, Poznan University of Physical Education, 61-871 Poznan, Poland; 2 Department of Gynaecology and Obstetrics, Minimally Invasive Operative Gynaecology, Subdepartment IA and IB, Poznan University of Medical Sciences, 61-758 Poznan, Poland; iubesc@poczta.onet.pl; 3 Faculty of Health, Catholic University, 034 01 Ružomberok, Slovakia; zuzana.hudakova@ku.sk; 4 College of Polytechnics, 583 01 Jihlava, Czech Republic; 5 Department of Mathematical and Statistical Methods, Poznan University of Life Science, 60-637 Poznan, Poland; idzi.siatkowski@up.poznan.pl; 6 Division of Gynaecology, Department of Gynaecology, Poznan University of Medical Sciences, 61-758 Poznan, Poland; witold.kedzia@poczta.onet.pl (W.K.); grajarz@o2.pl (G.J.-B.)

**Keywords:** visceral manipulation, endometriosis, pelvic organ prolapse, postural stability, quality of life

## Abstract

**Background**: Visceral manipulation is a soft tissue manual work technique that originates from and is classified within the Osteopathic Manipulative Technique (OMT), focusing on the fascial tissue mobilisation of the visceral system. Manual therapy on internal organs is based on anatomy, physiology and physics. **Methods:** Sixty women with endometriosis and pelvic organ prolapse, aged 41.5 ± 12.02, participated in the study. The women had manipulation therapy once a week for 5 weeks. The World Health Organisation Quality of Life BREF questionnaire and a postural stability assessment were administered before and after performing visceral manipulation. **Results:** The *p*-value = 0.0093 obtained in the group with prolapses and the *p*-value = 0.0001 in the group with endometriosis indicated that the applied visceral manipulation improved the women’s quality of life. No effect of therapy was observed on postural stability. **Conclusions:** Visceral manipulation improved the quality of life of both study groups of women. A significant difference was also observed when comparing the two groups for area.

## 1. Introduction

Visceral manipulation is a soft tissue manual work technique that originated from and is classified within the Osteopathic Manipulative Technique, focusing on the mobilisation of the fascial tissues of the visceral system [1,2]. Gynaecological conditions should be considered as related to somatic dysfunction (SD) [3], which is defined as an altered function that is associated with inflammation and palpable symptoms in body structures in different regions of the body [4,5].

Manual therapy on internal organs is based on anatomy, physiology and physics, the study of which has provided the following information. (1) Visceral structures have a significant mass and are subject to the laws of physics just like the musculoskeletal system [6,7]. (2) Internal organs are connected to each other through a two-layered system of serous membranes, i.e., the pleurae, pericardium and peritoneum [8,9]. (3) Visceral structures are connected to each other through an extensive network of fascial connections [10,11,12,13]. (4) Nociceptive stimuli direct pain to the somatic system via the visceral reflex. The homeostatic afferent pathway is a sensory adjunct of the autonomic nervous system that carries signals from the primary small-diameter Adelta and C afferent fibres, representing the physiological state of each tissue of the body [14,15]. Visceral nociceptors are activated by ischaemia and inflammation [16,17,18]. (5) Intestinal wall contraction is regulated by a rich neuronal network that functions independently of the central nervous system [19]. (6) Visceral dysfunction, e.g., inflammation, contributes to central sensitisation and chronic pain states [20,21]. (7) Abdominal and thoracic wall scarring resulting from surgical procedures affects the soft tissues. Loss of mobility runs from the epidermis through subcutaneous tissue to muscular and visceral structures, which can contribute to local tissue dysfunction and pain conditions [13,22]. (8) Adhesions occur following trauma, surgery, tumours and inflammatory processes [23,24]. Adhesions are innervated and are capable of generating pain. They may contribute to constipation, small bowel obstruction, pelvic pain and infertility [25,26].

Visceral manipulation aims to improve or regain the mobility of internal organs [27]. Visceral manual therapy takes into account the three-dimensional dynamics of human body biomechanics, including musculoskeletal and musculo-fascial structures, the connective tissues of organs and reflex activities in the central and peripheral nervous system, including circulation and fluid drainage in the human body [28].

The internal organs, the muscular system and all other systems and structures in the human body must be understood in terms of the connective tissue that surrounds them and the body fluids that flow through them, i.e., blood and lymph [29]. In order to improve the health and how the human body functions, it is necessary to consider the effects on the aforementioned structures and especially the surrounding connective tissue and body fluids (blood and lymph) [29]. Andrew Taylor Still, who is the founder of osteopathy, believed that the cause of dysfunction/disease in the human body is an impaired circulation of body fluids [30]. In the case of internal organs, visceral dysfunction is said to be defined as the impaired mobility of the visceral structure and all associated muscular, neurological, vascular, lymphatic and bony components [31].

Endometriosis, i.e., the presence of endometrial tissue called the endometrium outside the uterine cavity, and pelvic organ prolapse are conditions that negatively affect women’s quality of life. This is particularly true in terms of their sexual life, social relationships and work [32,33]. The most common treatments for endometriosis are hormone therapy, physical medicine treatments and diet [34,35,36]. Pelvic floor muscle strengthening exercises are also used to treat pelvic organ prolapse [37]. Organ prolapse is often accompanied by urinary incontinence [36]. Depending on medical indications, both conditions are treated surgically [38,39].

Abnormalities in muscle and fascial tone in the pelvic region can affect postural stability [13]. Postural stability refers to the ability to hold the body in a position to perform a task successfully. Postural stability requires adequate postural muscle strength and neuromotor control. Pelvic floor dysfunction leads to abnormalities in the pelvic–lumbar complex, affecting stability and posture [13,40]. Correct posture and abdominal muscle tone affect normal pelvic floor muscle tone, which in turn affects urinary continence [41]. Patients with endometriosis often adopt a flexed position of their body (they have a deepened thoracic kyphosis of the spine) due to pain, which in turn influences the adoption of an abnormal body posture [42]. With a view to demonstrating the practical use of visceral manipulation, it was decided to test whether this therapy could have an impact on the health of women diagnosed with endometriosis and pelvic floor static disorders. A review by Ruffini et al. provides information on the use of OMT for somatic dysfunction in patients with endometriosis [3]. Although the authors did not include details about the specific treatment techniques used in the included studies, analysing these studies allows for some insights into the osteopathic approaches applied in research within this clinical context. For example, in the case report from Goyal et al. [43], it is noted that the osteopathic treatment consists of all the major diaphragms’ release (pelvic diaphragm, abdominal diaphragm, thoracic outlet and hyoid diaphragm release) during the first session and, in the second session, gastro-oesophageal junction release, sigmoid colon release, cranial therapy to the occiput, sacral release and dural tube rocking. In the pilot study from Sillem et al. [44], it is reported that a standardised approach was used for osteopathic treatment starting with release of musculoskeletal blocks, particularly of the sacroiliac joints. Depending on clinical findings, this was followed by the mobilisation of the diaphragm and abdominal organs using standard techniques. The pelvic floor was released using the so-called grand manoeuvre: the movement of the abdominal organ compartment in a cranial direction. The treatment was concluded in many cases with the mobilisation of the temporomandibular joints and cervical spine. Each patient was prescribed six treatment sessions, of which a median of six were carried out, usually at weekly intervals. Ott presented a case study of a 24-year-old woman who was diagnosed with endometriosis and started hormone therapy. As there were complaints of LBP (lower back pain), pelvic and lower abdominal pain, osteopathic treatment was applied, based on a model based on the patient’s needs. The osteopath applied four sessions of OMT, which had a significant effect on reducing the pain symptoms, so that the hormone therapy started was discontinued after an agreement with the doctor [45]. Daraï and colleagues [46] studied the effect of standardised osteopathic treatment on women with colorectal endometriosis. This study demonstrated the effect of osteopathic treatment in improving women’s quality of life. Another study by Daraï et al. found in women with endometriosis that a single OMT session had a positive effect on pain reduction and led to an improvement in their general health, social functioning and mental health [47].

Prior to the study, the research hypothesis was defined: visceral manipulation applied for 5 weeks (one time per week) has an effect on postural stability parameters and the quality of life of women with pelvic organ prolapse and endometriosis.

## 2. Materials and Methods

### 2.1. Type of Study and Participants

Sixty women (n = 30) with endometriosis and n = 30 with pelvic organ prolapse, aged 41.5 ± 12.02, participated in the study. Recruitment of the women participating in the study was conducted over a one-year period. At the time of recruitment, women were interviewed with regard to the study’s inclusion and exclusion criteria. Participants in the study were women who self-referred for osteopathic treatment. Inclusion criteria were as follows: use of surgical treatment only, use of visceral manipulation at the latest one month after surgical treatment, no history of physiotherapy or osteopathy, no concomitant medical conditions (e.g., diabetes, hypertension), no medication, no stimulants or depressants used (e.g., drinking alcohol or smoking) and written consent provided to participate in the study in accordance with the Declaration of Helsinki. Exclusion criteria were as follows: failure to meet the inclusion criterion, inability to attend a visceral therapy session, e.g., due to infection, and lack of written consent to participate in the study. Prior to the visceral manipulation being performed, study participants were informed how it was to be performed and that they could opt out at any stage of the study. Participation in the study was entirely voluntary and the visceral manipulation was performed free of charge.

### 2.2. Visceral Manipulation

Visceral manipulation was performed between 3.00 p.m. and 8.00 p.m. in a warm and quiet room on women with endometriosis and pelvic organ prolapse after surgical treatment. During the visceral manipulation, the study participant lay on her back in her underwear on an adjustable therapy table. Visceral manipulation was performed on all study participants by the same therapist and was performed one time per week for 5 weeks. The following procedures were performed: diaphragm muscle relaxation, a general technique to relax the pelvic diaphragm and urogenital diaphragm, stretching of the pubococcygeal ligament, relaxation of the curtain membrane, a general technique to relax the uterus, a general technique to relax the ovaries and fallopian tubes, and mobilisation of the uterine broad ligament [48]. Stretching pressure lasting 10 s was applied during visceral manipulation and repeated until the patient felt no soft tissue stretching. The duration of the session for each patient was 20 min, timed using a stopwatch. Visceral manipulation was performed by only one person, an experienced physiotherapist with twenty-eight years of experience, and a certified osteopath with six years of experience. In the event of a study woman being unable to attend a session, e.g., due to an infection, visceral therapy was not rescheduled and the woman in question was excluded from further participation in the study. As the proposed number of sessions was five, one per week, we structured the sessions with a fixed schedule on designated days of the week to ensure a weekly interval was maintained, thus ensuring a fixed protocol for all participants in the study.

### 2.3. Outcome Measures: Quality of Life and Postural Stability

The study used the standardised World Health Organisation Quality of Life BREF (WHOQOL-BREF) questionnaire. On the day of visceral manipulation, the women completed a questionnaire anonymously before it was performed. On the last day, post-session, the participants also completed a questionnaire anonymously. For the purpose of this publication, the individual domains of the questionnaire were not analysed. The maximum number of points that the respondent could receive was 130 and the minimum was 26 points.

Postural stability was also evaluated before the visceral therapy session and after the last session. Postural stability (anterior–posterior, lateral–medial pivoting and postural field) was assessed using the Computerised Stability Platform CQStab2P-vUSB-1506. The test was conducted with the participants’ eyes open for 30 s. The subject stood on the platform barefoot, with the upper limbs along the torso and looking ahead. After the test was completed, a report of postural stability parameters was produced in the device’s computer programme. The values of anterior–posterior and lateral–posterior swings and the ellipse area of the centre of gravity were taken into account for statistical analysis. The smaller the values for excursion and ellipse area, the better the postural stability. Individual manufacturers of stabilometric platforms do not provide standards for healthy or sick people. The outcome assessor was not aware of the aim of the study.

### 2.4. Statistical Analysis

The data collected were statistically analysed. As a normal distribution of numbers was not observed, the Wilcoxon test was applied to test whether visceral manipulation has a significant effect on improving quality of life and postural stability values of the groups of women studied. A comparison was also made between the group of women with endometriosis and the group of patients with pelvic organ prolapse as to whether visceral manipulation affected quality of life and postural stability values in the study groups, also using the Wilcoxon test. The results are also presented as boxplots. The R statistical package [49] was used for this analysis. The statistical analysis was carried out by an experienced mathematician and statistician. The study was carried out after the approval of the Bioethics Committee of Poznan University of Medical Sciences No. 305/23 and after written consent of the study participants was obtained. In addition, the study was registered in the Clinical Trials Registry (Registry ID: NCT05978414).

## 3. Results

A total of 96 women with surgical treatment were enrolled in the study (42 with endometriosis and 54 with pelvic organ prolapse), of whom 30 were excluded because they did not meet the criteria for participation. A total of 66 women started the study. During its course, three women (with endometriosis) were excluded because they could not attend the scheduled visceral manipulation session and three women (with pelvic organ prolapse) dropped out on their own accord after the first visceral therapy session. After the visceral manipulation sessions, the women reported no side effects.

**Figure 1:**
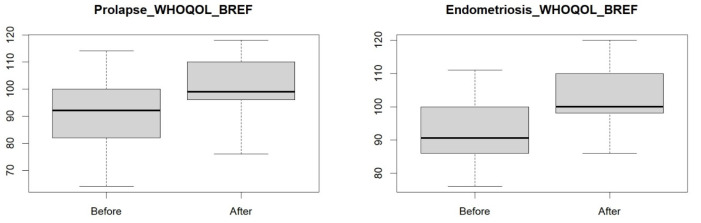
Quality of life in a group of women with endometriosis and pelvic organ prolapse before and after visceral manipulation.

Before proceeding to the statistical analysis, a check was made to see if there was a normal distribution of numbers. Since it was not observed, the Wilcoxon test was used to determine whether the visceral manipulation improved the quality of life of the women with endometriosis and pelvic organ prolapse. The *p*-value = 0.0093 (Table 1, Figure 1) obtained in the group of women with pelvic organ prolapse and the *p*-value = 0.0001 (Table 1, Figure 1) in the group of women with endometriosis indicate that the applied visceral manipulation improved the quality of life of the women.

**Figure 2:**
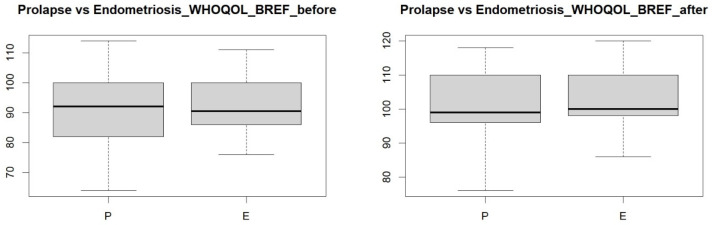
Comparison of quality of life between groups of women with endometriosis and pelvic organ prolapse before and after visceral therapy.

Next, a comparison was made between the study groups of women with endometriosis and pelvic organ prolapse before the visceral manipulation to determine whether the study groups differed. The resulting *p*-value = 0.9882 (Figure 2) indicates that the study groups did not differ, i.e., both study groups had a similar quality of life. Also, after the visceral manipulation, the two groups did not differ in terms of *p*-value = 0.2254 (Figure 2), meaning that after the therapy, both groups of women had a similar quality of life.

The next step in the statistical analysis was to see if the visceral manipulation influenced postural stability values. For this group of data, a normal distribution was not observed either; thus, the Wilcoxon test was applied to determine whether the visceral manipulation affected the values of postural stability in the group of women with pelvic organ prolapse and endometriosis. The Wilcoxon test was also used to make comparisons between the study groups of women. The results are presented as boxplots.

**Figure 3:**
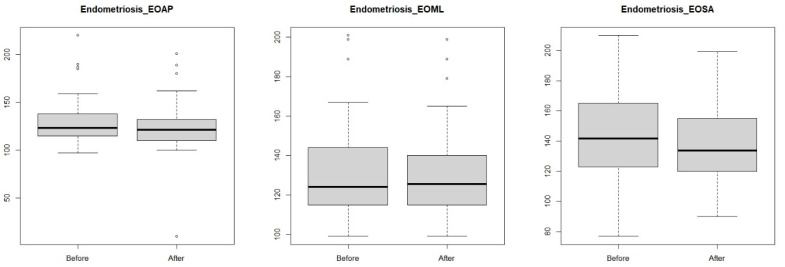
Anterior–posterior (eyes-open anterior–posterior) and lateral–medial (eyes-open medial–lateral) outcrops and surface area (eyes-open surface area) in a group of women with endometriosis before and after visceral manipulation.

**Figure 4:**
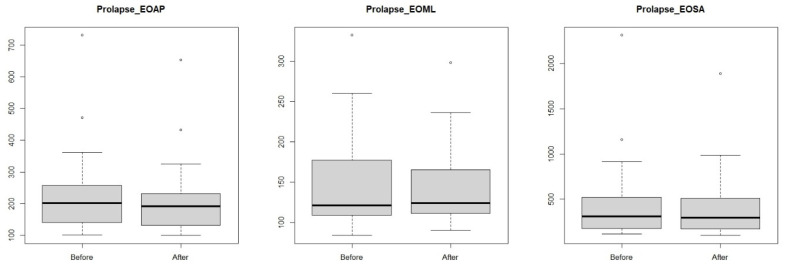
Anterior–posterior (EOAP: eyes-open anterior–posterior) and lateral–medial (eyes-open medial–lateral) outcrops and surface area (eyes-open surface area) in a group of women pelvic organ prolapse before and after visceral manipulation.

The values obtained for postural stability showed no effect of the visceral manipulation in both study groups of women with their eyes open for anterior–posterior and lateral–medial tilt and area (Table 2, Figure 3 and Figure 4).

**Figure 5:**
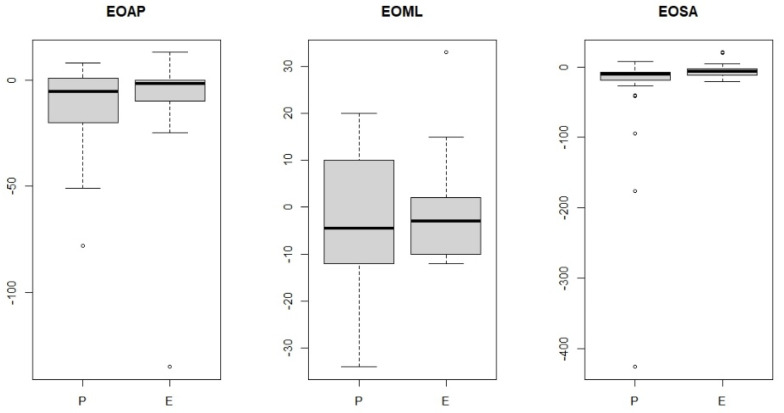
Comparisons of endometriosis (E) vs. pelvic organ prolapse (P) groups for anterior–posterior pivoting (EOAP: eyes-open anterior–posterior), lateral–medial pivoting (EOML: eyes-open medial–lateral) and surface area (EOSA: eyes-open surface area).

A comparison was then made between the study groups of women with endometriosis and pelvic organ prolapse. No significant statistical value was observed between the study groups for anterior–posterior and lateral–medial pivoting (Table 3, Figure 5), meaning that these values were similar in the two study groups after the visceral manipulation. A significant statistical value was observed for the area after the visceral manipulation, which means that the study groups differed (Figure 5).

## 4. Discussion

The World Health Organisation (WHO) defines quality of life as a person’s perception of their position in life in the context of the culture and value system in which they live, in relation to their goals, expectations, standards and concerns [43]. A cross-culturally applicable quality of life assessment was developed by the WHOQOL group with the support of fifteen international field centres [50].

Gynaecological conditions are common in women worldwide, carrying high treatment costs [3]. Worldwide, it is estimated that 10% of women of reproductive age have endometriosis [51], and pelvic organ prolapse is experienced by 40% of women and is predicted to become increasingly frequent [52].

The use of OMT in gynaecological conditions can support neurophysiological and cerebral activity in healing processes [3,53,54,55,56]. In visceral manipulation, the osteopath’s hands are the main tool for working with the patient, meaning interoception through the sense of touch can play a key role in this therapy [55]. Touch has been identified as an exteroceptive and interoceptive modality [55]. Interoception is supported by small-diameter low-conductance unmyelinated C-tactile (CT) fibres, which may be significant in the mechanism of action of touch-based manual therapy [55]. Manual therapies, which include visceral manipulation, still do not have a well-founded clinical value [55]. Performing visceral gynaecological techniques also involves working within the abdominal cavity, so it would also be reasonable to consider mechanisms occurring in the gastrointestinal (GI) tract, which has excellent neural control at two levels [57]. One is the enteric nervous system (ENS), comprising Auerbach’s and Meissner’s plexuses, described as a local nervous mechanism that controls bowel function independently, and the second level is the innervation coming from the central nervous system (CNS) [57]. Perhaps the positive effect of visceral manipulation should also be sought in the neural pathways transmitting intestinal information to the CSN [58]. Consideration should also be given to the occurrence of viscero-somatic reflexes, which appear when the diseased visceral organ transmits information via afferent nerves [59]. The spinal cord supervises somatic efferent and afferent stimulation and visceral afferent stimulation at the same segmental level via the autonomic nervous system. A high degree of somatic and visceral convergence is present in the area of lamina I of grey matter. At the interneurons of lamina I, somatic fibres and visceral C-fibres form synapses. The stimulation of these interneurons produces a sympathetic signal to internal organs with dysfunction [60]. Abnormal activity of the nervous system at given spinal levels creates a maladaptive reflex arc that also contributes to internal organ dysfunction [60]. Osteopaths use OMT to attenuate sympathetic nervous system activity and, at the same time, visceral dysfunction, through the somato-peritoneal reflex network. Regardless of the technique used, the aim of osteopathic manipulative treatment is to eliminate or reduce somatic dysfunction in order to decrease the somatic component in the spinal cord and improve the overall functioning of the nervous system [60].

Our study results indicate that visceral manipulation can be an effective tool to improve the quality of life of women with endometriosis or pelvic organ prolapse. It is worth emphasising that this is a non-pharmacological and non-invasive way to improve quality of life. In our study, we used the standardised World Health Organisation Quality of Life BREF questionnaire (WHOQOL-BREF).

De Marco et al. used visceral manipulation in their randomised control trial (RCT) in women with urinary incontinence. In addition to visceral manipulation, patients received pelvic floor muscle training, and thoracic spine mobilisation was introduced [61]. When performing visceral manipulation, tensile pressure lasting 5 s was applied. The authors showed no positive effect of the combination of therapies in women on the reduction in incontinence symptoms. The International Consultation on Incontinence Questionnaire-Urinary Incontinence Short Form (ICQ-UI-SF) was used in this study. In this study, the patients underwent visceral manipulation one time per week for 5 weeks, and we also used visceral manipulation one time per week for 5 weeks.

Yosri et al., in an RCT, used visceral manipulation in combination with a low-calorie diet in women with polycystic ovary syndrome accompanied by menstrual problems. This study found a statistically significant improvement in the reduction in menstrual problems in the group in which visceral manipulation was used [62]. In this study, tensile pressure lasting 6 s was applied and repeated until the patient did not feel any stretching of the soft tissues. The Polycystic Ovary Syndrome Quality of Life scale (PCOSQOL) questionnaire was used to determine symptoms associated with menstrual disorders.

In their RCT, Lagrange et al. used visceral manipulation in post-surgical breast cancer patients who were undergoing chemotherapy to alleviate side-effects such as vomiting, nausea and constipation. In this study, no significant differences were found for the reduction in vomiting, nausea and constipation. In contrast, patient-reported quality of life related to digestion improved significantly [63]. The Cancer (EORTC) QLQ-C30 Quality of Life questionnaire was used to assess quality of life in this study.

Visceral manipulation has also been used in patients with musculoskeletal dysfunction. Tamer et al. used visceral manipulation in patients with non-specific lower back pain. In their RCT, they used the 36-Item Short Form Survey (SF-36) Quality of Life questionnaire. The authors showed a positive effect of visceral manipulation on improving the quality of life of non-specific LBP sufferers, demonstrating the effect of therapy on reducing pain sensations among the subjects [64]. Moreover, Panagopoulos et al. conducted an RCT to evaluate the effects of standard physiotherapy and visceral manipulation in patients with sacral pain. Their results showed that the intervention group experienced less pain than the placebo group after 52 weeks [65].

Eguara et al. applied visceral manipulation to patients with gastroesophageal reflux disease (GERD). They used the GerdQ questionnaire and the Cervical Range of Motion (CROM) to assess the effectiveness of the therapy. The results of the study showed alleviation of GERD symptoms and cervical spine mobility after visceral manipulation [66]. In this study, patients received only two therapy sessions with a one-week interval between the first and second session.

Neto et al.’s RCT showed a positive effect of visceral manipulation in post-stroke patients in terms of reducing constipation. Due to the nature of their condition, participants in the study were also subjected to neurological physiotherapy for motor improvement. In this study, the mobilisation of the ascending colon, descending colon, sigmoid colon and sphincters (cardiac, pyloric, Oddi, duodenogastric and ileocaecal) was performed. Stretching pressure for 1 min was applied [67]. The authors also assessed the postural stability of the patients, observing improved parameters in the group that underwent visceral manipulation [67].

Postural stability is most commonly assessed in people with musculoskeletal conditions [68,69] or in neurological diseases [70,71]. Previous studies on the postural stability and quality of life of women have examined assessments related to lower back pain, mineral density, muscle strength, chemotherapy and the impact of physical activity in women with breast cancer [72,73,74,75]. Buscemi et al.’s study was carried out on healthy subjects but showed an effect of OMT, which improved postural stability values [76].

Our results for postural stability showed in the comparison of the female groups studied that they differed statistically significantly for the value of the area of the centre of gravity, which may indicate the effect of the visceral manipulation, which improved their stability. The surface area is the area in which the body’s centre of gravity oscillates. The smaller the surface area, the better the body’s balance. In an adult, the centre of gravity in the pelvis is located at the level of the first sacral vertebra—S1 [77]. In turn, the pelvis acts as a hinge between the spine and hips to maintain balance during bipedalism [77]. Pelvic organ prolapse in women affects the pelvic area, and the ongoing disease process of endometriosis also predominantly affects the pelvis. It may be the case that the ongoing disease process affects the location of the centre of gravity in women.

OMT techniques can have different effects on neurophysiological function and should be promoted in a personalized fashion by focusing on somatic dysfunction (SD) [4,78]. In practice, osteopaths use many different techniques on different parts of the body during the same session [78]. The black box approach should also be kept in mind [78]. The black box leaves the osteopath free to apply the main principles of osteopathy, treating ‘what he finds’ in the patient, while intermediate parameters are assessed to measure outcomes [78].

Visceral manipulation was used in a standardised way to reinforce the methodology and the possibility of generalising the results. In our protocol, we assumed that during each session, the same visceral manipulation would be performed for all patients lasting the same amount of time. The intervention protocol we adopted was also intended to make our study better understood by other professionals who do not refer to somatic dysfunction in their practice, which is a characteristic term for osteopaths and refers to distinctive strategies. Osteopathic treatment must focus on the lowering of somatic dysfunction through shared decision making by the therapeutic team formed by the doctor and the osteopath. In this case, the selection of the appropriate osteopathic treatment will also depend on the professional experience of the doctor and osteopath. In addition, everyday osteopathic praxis, in which osteopathic therapy is patient-centred, should be incorporated and involve outcome-based strategies. Osteopathic treatment aims to improve patients’ adaptability by focusing on somatic dysfunction through shared decision making by the therapeutic team formed by osteopaths and other health professionals. The tool of osteopaths to work with patients is their hands. Touch is a tool for detecting somatic dysfunctions like neurofascial active areas, which can act as an osteopath–patient interface to transmit the biological and physiological effects of touch [79,80]. Inhibition tests, which are manual functional tests used in the diagnosis of SD, are tests in which the osteopath applies manual mechanical stimuli to dysfunctional tissues and assesses the biological responses that occur simultaneously with the application of the stimulus [81]. Osteopathic structural–functional models, which include the biomechanical, respiratory–circulatory, metabolic, neurological and biopsychosocial models, have been developed to promote our understanding of patients’ complex health functioning. The models also contribute to the preparation of a comprehensive management plan to restore and maintain their health potential [82].

The use of standardized osteopathic visceral techniques, selected based on the expertise of the involved professionals (i.e., the physician and the osteopath), differs significantly from real-world daily osteopathic practice. In routine clinical settings, practitioners typically adopt a whole-body personalized osteopathic approach. This approach involves a shared decision-making process that incorporates the patient’s input, tailoring the intervention to the individual’s clinical presentation [83].

### Strengths and Limitations

One definite limitation of this study is the small group of women surveyed; thus, the results obtained should be treated as preliminary observations. Certainly, one limitation of this study is also the lack of groups of women who could be treated with a placebo or women who received conservative treatment. On the other hand, the strengths of this study are the inclusion criterion for women less than one month after surgical treatment and the rigid protocol of conducting five therapy sessions with a weekly interval between sessions and with a fixed time for each session. Further research taking into account both the weaknesses and strengths of this study will provide hard evidence for a greater demonstration of the positive effects of visceral manipulation in women with endometriosis and pelvic organ prolapse.

## 5. Conclusions

The use of five treatments at weekly intervals improved the quality of life of women with endometriosis and pelvic organ prolapse. Visceral manipulation also improved the area of the centre of gravity in both study groups.

## Figures and Tables

**Table 1:** Quality of life of women with endometriosis and pelvic organ prolapse before and after visceral manipulation.

Group	*p*-Value
endometriosis WHOQOL BREF	0.0001
pelvic organ prolapse WHOQOL BREF	0.0093

**Table 2:** Postural stability values of a group of women with endometriosis and pelvic organ prolapse with their eyes open for anterior–posterior pivot (EOAP: eyes-open anterior–posterior), lateral–medial pivot (EOML: eyes-open medial–lateral) and surface area (EOSA: eyes-open surface area).

Group	*p*-Value
endomertiosis EOAP	0.4158
endometriosis EOML	0.9234
endometriosis EOSA	0.3827
pelvic organ prolapse EOAP	0.6152
pelvic organ prolapse EOML	0.9117
pelvic organ prolapse EOSA	0.6843

**Table 3:** Comparison between study groups of women with endometriosis and pelvic organ prolapse in terms of their postural stability values with their eyes open for anterior–posterior pivoting (EOAP: eyes-open anterior–posterior), lateral–medial pivoting (EOML: eyes open medial–lateral) and surface area (EOSA: eyes-open surface area).

Group	*p*-Value
endometriosis vs. pelvic organ prolapse EOAP	0.558
endometriosis vs. pelvic organ prolapse EOML	0.5385
endometriosis vs. pelvic organ prolapse EOSA	0.0221

## Data Availability

Data are available upon request.
